# ^1^H R_1ρ_ relaxation dispersion experiments in aromatic side chains

**DOI:** 10.1007/s10858-021-00382-w

**Published:** 2021-09-12

**Authors:** Matthias Dreydoppel, Roman J. Lichtenecker, Mikael Akke, Ulrich Weininger

**Affiliations:** 1 Institute of Physics, Biophysics, Martin-Luther-University Halle-Wittenberg, 06120 Halle (Saale), Germany; 2 Institute of Organic Chemistry, University of Vienna, 1090 Vienna, Austria; 3 Division of Biophysical Chemistry, Center for Molecular Protein Science, Department of Chemistry, Lund University, P.O. Box 124, 22100 Lund, Sweden

**Keywords:** Conformational exchange, Protein dynamics, Aromatic side chains, Rotating-frame relaxation, Aromatic ring-flip

## Abstract

Aromatic side chains are attractive probes of protein dynamic, since they are often key residues in enzyme active sites and protein binding sites. Dynamic processes on microsecond to millisecond timescales can be studied by relaxation dispersion experiments that attenuate conformational exchange contributions to the transverse relaxation rate by varying the refocusing frequency of applied radio-frequency fields implemented as either CPMG pulse trains or continuous spin-lock periods. Here we present an aromatic ^1^H *R*_1*ρ*_ relaxation dispersion experiment enabling studies of two to three times faster exchange processes than achievable by existing experiments for aromatic side chains. We show that site-specific isotope labeling schemes generating isolated ^1^H–^13^C spin pairs with vicinal ^2^H–^12^C moieties are necessary to avoid anomalous relaxation dispersion profiles caused by Hartmann–Hahn matching due to the ^3^*J*_HH_ couplings and limited chemical shift differences among ^1^H spins in phenylalanine, tyrosine and the six-ring moiety of tryptophan. This labeling pattern is sufficient in that remote protons do not cause additional complications. We validated the approach by measuring ring-flip kinetics in the small protein GB1. The determined rate constants, *k*_flip_, agree well with previous results from ^13^C *R*_1*ρ*_ relaxation dispersion experiments, and yield ^1^H chemical shift differences between the two sides of the ring in good agreement with values measured under slow-exchange conditions. The aromatic^1^H *R*_1*ρ*_ relaxation dispersion experiment in combination with the site-selective ^1^H–^13^C/^2^H–^12^C labeling scheme enable measurement of exchange rates up to *k*_ex_ = 2*k*_flip_ = 80,000 s^–1^, and serve as a useful complement to previously developed ^13^C-based methods.

## Introduction

Conformational dynamics in proteins on the microsecond to millisecond time scales are often linked to biological function (Mittermaier and Kay 2009). Transiently populated high-energy states play important roles in enzyme catalysis (Boehr et al. 2006; Cole and Loria 2002; Eisenmesser et al. 2002) or ligand binding by conformational selection (Demers and Mittermaier 2009; Malmendal et al. 1999). Such conformational transitions generally lead to a modulation of NMR parameters as the chemical shift (Gutowsky and Saika 1953), residual dipolar coupling (Igumenova et al. 2007; Vallurupalli et al. 2007) or strong scalar coupling (Weininger et al. 2013b). The conformational dynamics can be probed by NMR relaxation dispersion methods (Palmer 2004; Palmer et al. 1991), such as *R*_1*ρ*_ (Akke and Palmer 1996; James et al. 1977) or Carr-Purcell-Meiboom-Gill (CPMG) experiments (Carr and Purcell 1954; Loria et al. 1999a, 1999b; Meiboom and Gill 1958), and chemical exchange saturation transfer (CEST) methods (Forsen and Hoffman 1963; Palmer and Koss 2019; Vallurupalli et al. 2012, 2017) in the case of slower time scales.

Phenylalanine, tyrosine, histidine and tryptophan all have aromatic side chains, which make them an interesting subgroup of amino acids that serve multiple functions in proteins. Aromatic side chains are bulky and constitute a significant proportion of the protein hydrophobic core. They typically form pairs or clusters on the basis of specific aromatic-aromatic interactions (Burley and Petsko 1985, 1989). Furthermore, aromatic residues are overrepresented in protein binding interfaces, where they contribute a significant part of the binding free energy (Birtalan et al. 2010; Bogan and Thorn 1998; Lo Conte et al. 1999). Finally, tyrosine and especially histidine play critical roles in enzyme catalysis, where they make up 6% and 18%, respectively, of all catalytic residues (Bartlett et al. 2002). Histidine can exist in three different states, one protonated and two neutral tautomeric forms. Despite their generally tight packing, Phe and Tyr residues undergo intermittent 180° transitions (‘ring flips’) of the *χ*_2_ dihedral angle and thereby provide unique information of transient 'breathing' processes of proteins (Dreydoppel et al. 2020; Wagner 1980; Wagner et al. 1976; Weininger et al. 2014b). Each of these properties makes aromatic side chains highly interesting and powerful probes for studying protein dynamics.

Recent developments have enabled straightforward and robust site-selective ^13^C labeling of aromatic side chains (Kasinath et al. 2013; Lundström et al. 2007; Schörghuber et al. 2018; Teilum et al. 2006; Weininger 2019), which eliminate unwanted relaxation pathways and coherent magnetization transfer via one-bond ^13^C–^13^C couplings. These advancements have made possible advanced studies of protein dynamics involving aromatic side chains, including methods to characterize fast (ps–ns) timescale dynamics via ^13^C relaxation rate constants (Weininger et al. 2012a) that enable studies of order parameters (Boyer and Lee 2008; Kasinath et al. 2013, 2015). Furthermore, slower (µs–ms) timescale dynamics have been probed with ^13^C relaxation dispersion experiments, either using CPMG (Weininger et al. 2012b) or *R*_1*ρ*_ refocusing elements (Weininger et al. 2014a). In particular, these relaxation dispersion methods have been used to measure ring-flip rates (Dreydoppel et al. 2020; Weininger et al. 2014b) and transient histidine tautomerization (Weininger et al. 2017). Complementary ^1^H CPMG relaxation dispersion experiments are applicable to a subset of aromatic sites, namely His δ2, His ε1 and Trp δ1. Other positions are inaccessible due to sizeable ^3^*J*_HH_ couplings and possibly strong ^1^*J*_CC_, where ^1^* J*-couplings are equal or greater than the chemical shift difference, can cause severe artifacts (Raum et al. 2018). Site-selective ^1^H–^13^C/^2^H–^12^C labeling addresses both issues and allows to obtain artifact-free ^1^H CPMG relaxation dispersion profiles (Raum et al. 2019).

It is expected that ^1^H *R*_1*ρ*_ experiments make it possible to access faster time scales, since the attainable refocusing frequency scales with the gyromagnetic ratio and the RF field amplitude, *ω*_1_ = *γB*_1_. ^1^H *R*_1*ρ*_ experiments have successfully been applied to the aromatic position H8 of adenine and guanine and in RNA (Steiner et al. 2016), which is not affected by sizeable ^3^*J*_HH_ couplings or cross relaxation with remote protons. The situation is more challenging in certain aromatic protein residues, where ^3^*J*_HH_ is on the order of 7–8 Hz and the chemical shift difference between vicinal ring protons is often small. Here we demonstrate these problems can be solved by site-selective ^1^H–^13^C/^2^H–^12^C labeling, which is necessary and sufficient to ensure artifact-free ^1^H *R*_1*ρ*_ relaxation dispersion data for aromatic side chains in proteins.

## Materials and methods

### Protein samples

**Fig. 1:**
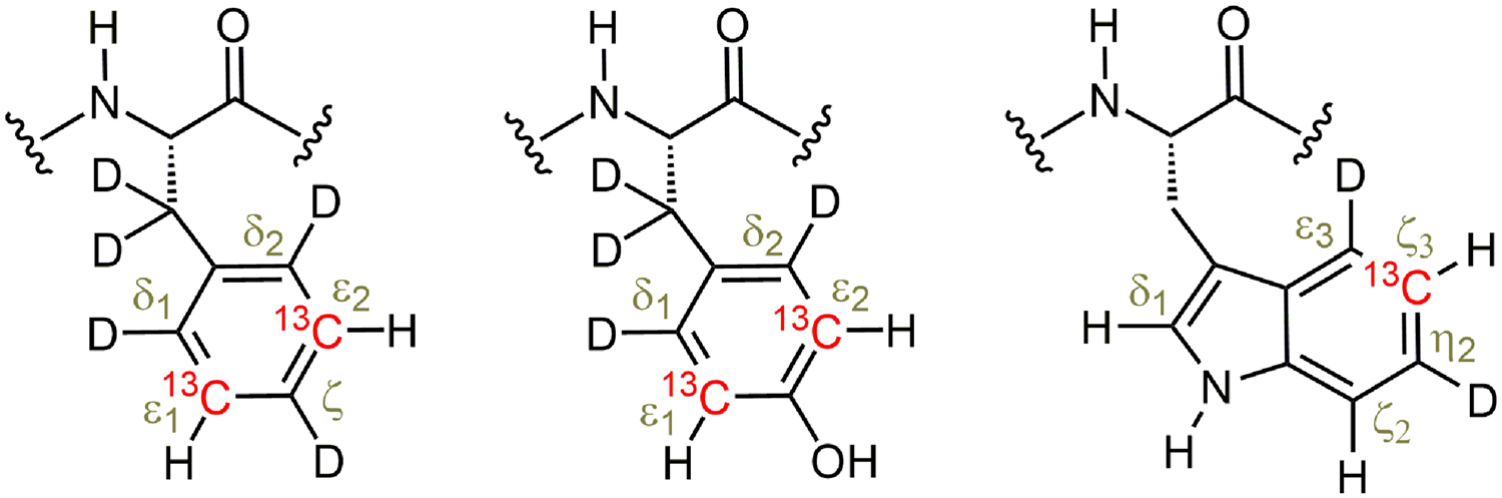
Labeling patterns in Phe, Tyr and Trp (from left to right), using site-selectively ^1^H/^2^H and ^13^C labeled -ketoacids as precursors (Lichtenecker 2014; Lichtenecker et al. 2013). Protons and deuterons are displayed by H and D, respectively. ^13^C is shown in red, other carbon positions are ^12^C

The B1 domain of the bacterial antibody-binding protein G (GB1) containing the mutations T2Q, N8D and N37D (QDD variant) was expressed and purified as described previously (Lindman et al. 2006), with three different labeling patterns: (i) site-selective ^13^C/^12^C using 2-^13^C_1_ glucose with natural abundance ^1^H incorporation (Lundström et al. 2007), (ii) site-selective ^1^H–^13^C/^2^H–^12^C labeling protein (Fig. 1) using site-selectively ^1^H/^2^H and ^13^C labeled -ketoacids as precursors (Lichtenecker 2014; Lichtenecker et al. 2013), 80 mg/L for Phe, 350 mg/L for Tyr, 10 mg/L for Trp, (iii) same as (ii) but with additional 70% background deuteration by expression in D_2_O (implemented only in the case of Phe ε*). NMR samples contained around 1 mM protein in 20 mM HEPES, 90% H_2_O/10% D_2_O with addition of small amounts of NaN_3_. The pH of the NMR samples was adjusted to 7.0.

### NMR spectroscopy

**Fig. 2:**
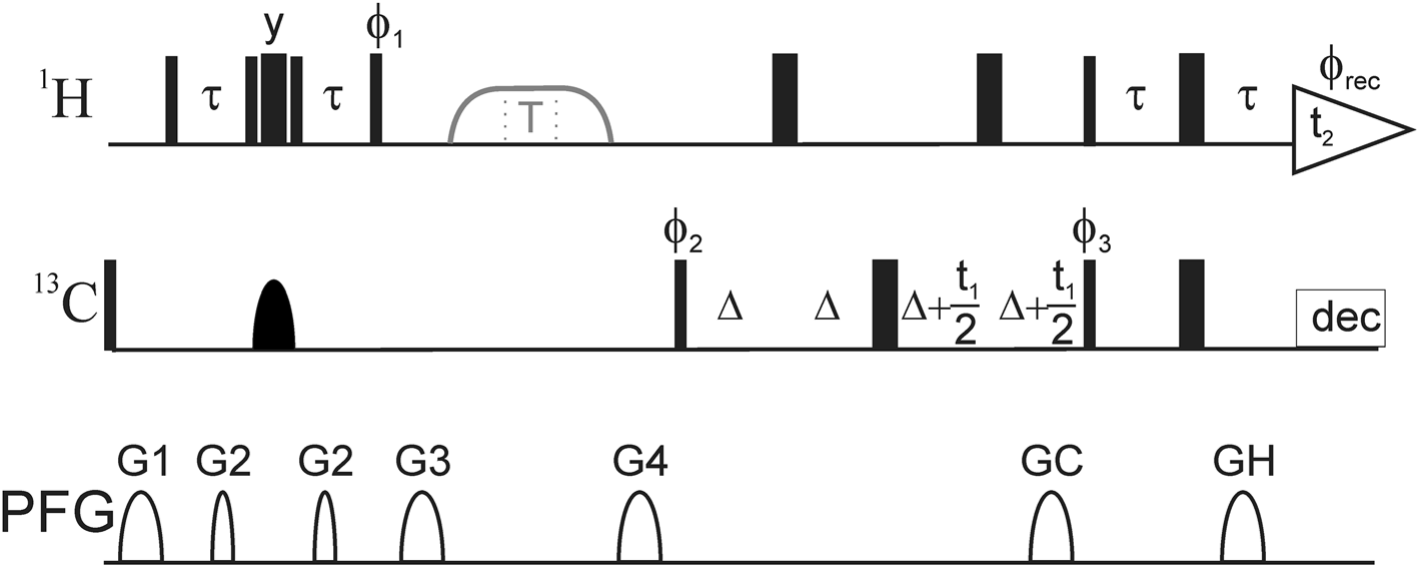
Pulse sequence for the ^1^H *R*_1*ρ*_ constant-time relaxation dispersion experiment for measuring conformational exchange of aromatic side chains in specifically ^1^H–^13^C/^2^H–^12^C labeled proteins. All pulses are applied along the *x*-axis unless otherwise indicated. Narrow (wide) solid bars indicate rectangular high-power 90° (180°) pulses. The continuous-wave spin-lock relaxation periods T and their flanking 4 ms tan/tanh adiabatic profiles (Mulder et al. 1998) are outlined in gray. The adiabatic sweep is initiated 25 kHz downfield or upfield of the spin-lock frequency. The wide semi-ellipse on ^13^C represents a REBURP (Geen and Freeman 1991) pulse with a bandwidth of 40 ppm. The pulse sequence can be modified to accommodate non-constant relaxation periods or off-resonance spin-locks by including a pair of ^13^C 180° pulses to mitigate the effects of dipole–dipole/CSA cross-correlated relaxation (Korzhnev et al. 2002; Massi et al. 2004). The delay τ can be set to 1.6 ms (Phe and Tyr), 1.35 ms (all aromatics) or 1.25 ms (His). The phase cycle is: ϕ_1_ = (4y, 4 − y), ϕ_2_ = (x, − x), ϕ_3_ = (x, x, − x, − x), ϕ_rec_ = (x, –x, –x, x, − x, x, x, − x). Pulsed field gradients G1–4 are employed to suppress unwanted coherences and artifacts, while GC and GH are encoding and decoding gradients, respectively, for echo/anti-echo coherence selection, obtained by inverting the signs of GH (Kay et al. 1992). The delay ∆ is equal to GC. For every second t_1_ increment ϕ_2_ and the receiver were incremented. Gradient durations (in ms) and relative power levels (in %) are set to (duration, power level) G1 = (1.0, 13), G2 = (0.5, 10), G3 = (1.0, 30), G4 = (1.0, 90), GC = (1.0, 80), GH = (1.0, -20.1)

All experiments were acquired on a Bruker Avance III spectrometer at a static magnetic field strength of 14.1 T, equipped with a room temperature probe. ^1^H *R*_1*ρ*_ relaxation dispersion experiments were performed using the pulse sequence shown in Fig. 2, with spectral widths of 14.0 ppm (^1^H) and 30.0 ppm (^13^C), by 1024 and 128 points, respectively. Nineteen experiments were performed at temperatures of 15, 20, 25, 30 and 35 °C. *R*_1*ρ*_ rate constants were measured in separate experiments conducted on-resonance with the signal of interest resulting in tilt angles of 90° from the z-axis, using a constant-time relaxation period (Mulder et al. 2001) of 20 ms and spin-lock field strengths varying between 1000 and 9000 Hz; the 4-ms adiabatic ramps used to align the magnetization along the effective spin-lock field do not achieve perfect alignment below 1000 Hz. Spectra were processed with NMRPipe (Delaglio et al. 1995) and analyzed with PINT (Ahlner et al. 2013).

### Data analysis

Relaxation rates in the rotating reference frame were calculated from the signal intensities measured in presence and absence of the spin-lock pulse, *I*(*ω*_1_) and *I*_0_, respectively, according to (Mulder et al. 2001)

1 $${R}_{1\rho }\left({\omega }_{1}\right)=-\frac{1}{{T}_{\text{SL}}}{\text{ln}}\frac{I\left({\omega }_{1}\right)}{{I}_{0}}$$

*R*_1*ρ*_ relaxation dispersions were fitted to the general equation for symmetric exchange derived by Miloushev and Palmer (2005) using fixed populations, *p*_1_ = *p*_2_ = 0.5, and treating ∆δ as a free parameter, which was compared to values measured under slow-exchange conditions at – 5 °C and 200 MPa. Data modeling utilized the Levenberg–Marquardt nonlinear least-squares optimization algorithm (Press et al. 2002) implemented in MATLAB. Errors in the fitted parameters were estimated using 1000 Monte-Carlo simulations per fit; the reported errors correspond to one standard deviation.

Activation parameters of the ring flips were determined by non-linear regression of the flip rates, *k*_flip_ = *k*_ex_/2, on the temperature *T*, using the Eyring equation. The Eyring equation was parameterized as

2 $${k}_{\mathrm{flip}}=\left(\frac{{k}_{\mathrm{B}}T}{h}\right)\times \mathrm{exp}\left[-(\Delta {H}^{\ddagger }-T\Delta {S}^{\ddagger })/RT\right]$$

where *k*_B_ and *h* denote Boltzmann’s and Planck’s constants, respectively, and ∆*H*^‡^ and ∆*S*^‡^ are the activation enthalpy and activation entropy, respectively.

### Hartmann–Hahn transfer calculations

A nucleus affected by a spin-lock radio frequency pulse with field strength ${\omega }_{1}=-\gamma {B}_{1}$ will perceive an effective field strength ${\omega }_{\mathrm{eff}}={({\Omega }^{2}+{{\omega }_{1}}^{2})}^{1/2}$ and will be oriented at an angle $\theta ={\mathrm{tan}}^{-1}({\omega }_{1}/\Omega )$ from the static magnetic field, with ${\Omega =\omega }_{0}{-\omega }_{c}$ denoting the offset between the nuclear precession frequency *ω*_0_ and the carrier frequency *ω*_c_ of the spin-lock pulse. In the case of two spins *I* and *S*, coupled by a scalar-coupling constant *J*, Hartmann–Hahn matching of the two effective fields causes magnetization transfer between them, according to the coherence transfer function given by (Brath et al. 2006; van de Ven 1995)

3 $${F}_{\mathrm{HaHa}}={A}_{\mathrm{HaHa}}{\mathrm{sin}}^{2}(D{T}_{\mathrm{SL}}/2)$$

where *T*_SL_ is the duration of the spin-lock period, $D={({\Delta }^{2}+ {{J}_{\mathrm{eff}}}^{2})}^{1/2}$, $\Delta ={\omega }_{\mathrm{eff},I}-{\omega }_{\mathrm{eff},S}$, ${J}_{\mathrm{eff}}=\frac{1}{2}J(1+\cos({\theta }_{I}-{\theta }_{S}))$ and *A*_HaHa_ is the coherence transfer amplitude

4 $${A}_{\mathrm{HaHa}}=\frac{1}{1+{(\Delta /{J}_{\mathrm{eff}})}^{2}}$$

Hartmann–Hahn mediated magnetization transfer from the monitored proton of the ^1^H-^13^C moiety to vicinal protons in the aromatic ring scales the intensity by a factor of 1 – *F*_HaHa_(*ω*_1_) for a given spin-lock period *T*_SL_. Consequently, the apparent *R*_1*ρ*_ rate constant is given by:

5 $${R}_{1\rho ,app}\left({\omega }_{1}\right)=-\frac{1}{{T}_{\text{SL}}}\left({\text{ln}}\frac{I\left({\omega }_{1}\right)}{{I}_{0}}+\mathrm{ln}\left(1-{F}_{\text{HaHa}}\left({\omega }_{1}\right)\right)\right)$$

where the first term describes the *R*_1*ρ*_ rate constant according to Eq. (1), i.e., in the absence of Hartmann–Hahn matching. Similarly, as a first approximation, in the case of *n* protons coupled to the monitored proton, the apparent rate constant is:

6 $${R}_{1\rho ,app}\left({\omega }_{1}\right)=-\frac{1}{{T}_{\text{SL}}}\left({\text{ln}}\frac{I\left({\omega }_{1}\right)}{{I}_{0}}+\sum_{i}^{n}\mathrm{ln}\left(1-{F}_{\text{HaHa,}i}\left({\omega }_{1}\right)\right)\right)$$

*A*_HaHa_ and *F*_HaHa_ were calculated for scalar-coupled protons in the aromatic rings of GB1 using *J* = ^3^*J*_HH_ = 7 Hz, *T*_SL_ = 20 ms, and the resonance frequencies measured in the spectrum acquired at 25 °C: 0.22 ppm between Y33 ε* and δ*; 0.65 ppm (0.16 ppm) between F52 ε* and δ*(ζ); and 0.98 ppm (0.13 ppm) between W43 ζ3 and ε3 (η2). In case of frequencies averaged by fast ring-flips, i.e. δ* and ε* in Phe and Tyr, it is sufficient to use them directly.

## Results and discussion

We performed aromatic ^1^H *R*_1*ρ*_ relaxation dispersion experiments on the small protein domain GB1, ^1^H–^13^C labeled in three different ways: (i) natural abundance protonation and site selective ^13^C/^12^C labeling; (ii) additional selective deuteration of vicinal hydrogen sites in order to eradicate ^3^*J*_HH_ couplings resulting in site-selective ^1^H–^13^C/^2^H–^12^C labeling (Fig. 1); or (iii) as (ii) with additional non-specific background deuteration at a level of 70%. There are five symmetric aromatic residues (Phe and Tyr) in GB1: Y3, F30, Y33, Y45 and F52. All but Y33 undergo relatively slow ring-flip processes that have been studied by aromatic ^13^C *R*_1*ρ*_ relaxation dispersion experiments (Dreydoppel et al. 2020); in particular Y3δ, Y3ε, F30δ, Y45ε and F52ε have been studied previously and provide valuable points of reference for comparison with our present work. Individual cross peaks from the two symmetric sites of the aromatic rings could be observed under slow exchange conditions (–5 °C, 200 MPa) for Y3δ, Y3ε, F30δ, F30ε and F52ε. Based on these results, the following ^13^C and ^1^H chemical shift differences could be extracted: Y3δ (2.11 ppm; 0.40 ppm), Y3ε (1.40 ppm; 0.50 ppm), F30δ (5.39 ppm; 0.84 ppm), F30ε (0.00 ppm; 0.56 ppm) and F52ε (1.76 ppm; 0.00 ppm). F52ε, which has a ^1^H chemical shift difference of zero, serves as a negative control in the present study, since it should not exhibit any exchange-mediated dependence of the *R*_1*ρ*_ rate constant on the spin-lock field strength. In addition, Y33ε, which is solvent exposed and undergoing very fast ring flips, is also expected to show an essentially flat dispersion profile. Furthermore, the two labeled sites in tryptophan, W43δ1 and W43ζ3 also serve as useful controls: W43δ1 does not experience sizeable ^3^*J*_HH_ couplings, and neither site undergoes exchange, since this residue does not readily undergo ring flips. In a previous study, site-selective ^1^H–^13^C/^2^H–^12^C labeling resulted in 99% correct labeling in case of Phe ε* and 75% in case of Trp ζ3, whereas Tyr ε* labeling yielded only 4% incorporation for reasons that are not clear (Raum et al. 2019). Here, we increased the amount of tyrosine precursor fourfold in the protein expression medium, resulting in 17% of the desired labeling for Tyr ε*, while the remaining 83% have natural abundance isotope incorporation. Therefore, 0.9% of the sample is ^13^C labeled at Tyr ε and protonated at the δ position. Thus, in the present sample the latter isotopomer is diluted by a factor of 20 compared to the desired one, which is sufficient to suppress ^3^*J*_HH_ coupling artifacts.

### Deuteration of vicinal protons is necessary in order to achieve artifact-free aromatic ^1^H R_1ρ_ relaxation dispersion profiles

**Fig. 3:**
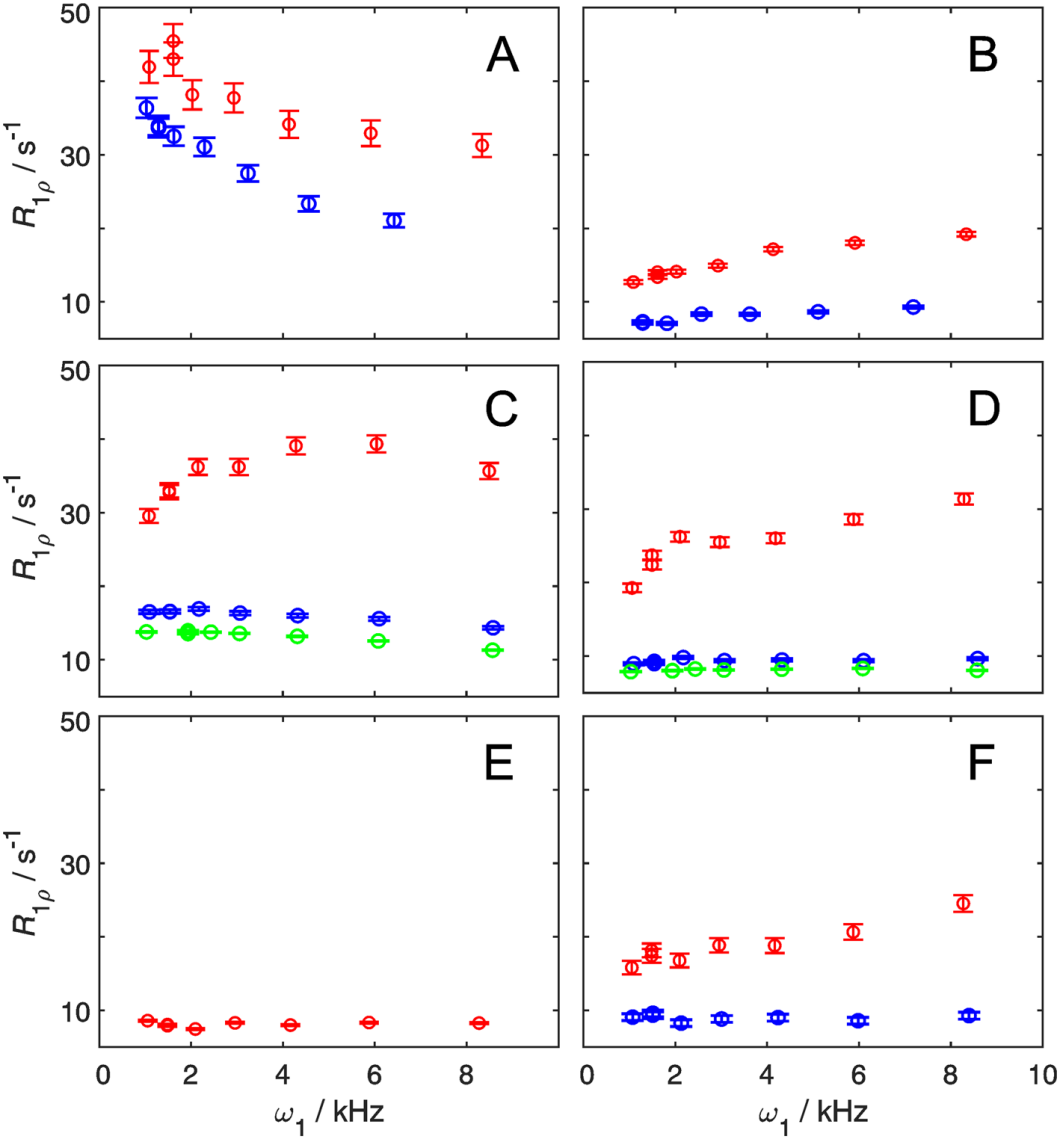
Aromatic ^1^H *R*_1*ρ*_ relaxation dispersions recorded on-resonance (tilt angle *θ* = 90° from the z-axis) at a static magnetic field-strength of 14.1 T and a temperature of 25 °C. Dispersion profiles obtained from uniformly protonated and site-selectively ^13^C/^12^C labeled samples are shown in red, from site-selectively ^1^H–^13^C/^2^H–^12^C labeled samples in blue, and site-selectively ^1^H–^13^C/^2^H–^12^C labeled samples with additional 70% background deuteration in green. Dispersion profiles are shown for Y3ε (**A**), Y33ε (**B**), F30ε (**C**), F52ε (**D**), W43δ1 (**E**) and W43ζ3 (**F**)

Figure 3 shows the resulting ^1^H *R*_1*ρ*_ relaxation dispersion profiles for the ^1^H–^13^C labeled ε sites of residues Y3, Y33, F30, and F52 (a-d), and for the δ1 and ζ3 sites of W43 (e–f). There is a notable variation among the dispersion profiles of the different residues and between the two^1^H/^2^H labeling patterns for each residue. The non-deuterated samples of residues Y33ε, F30ε, F52ε, and W43ζ3 (Figs. 3b–d and f; red symbols) all exhibit anomalous, ‘inverted’ relaxation dispersion profiles characteristic of sizeable (7–8 Hz) ^3^*J*_HH_ coupling (Raum et al. 2018). In contrast, the dispersion profile of Y3ε (Fig. 3a) does not appear anomalous, because the effect of Hartmann–Hahn matching is in part masked by a substantial chemical exchange contribution. In general, the magnitude of the artifact is more pronounced in the case of Phe (Fig. 3c, d) compared to Tyr (Fig. 3a, b), because the investigated ^1^Hε spins in Phe couple to two vicinal proton spins (δ and ζ) instead of one (δ) in Tyr. In the case of Trp ζ3, the magnitude is closer to the Tyr case. In the non-deuterated sample, W43δ1 displays a flat relaxation dispersion profile (Fig. 3e), as a result of its small coupling constant, ^3^*J*_HH_ < 2 Hz, and absence of exchange. This result is in keeping with the expectation outlined above regarding the role of W43δ1 as a negative control.

Site-selective deuteration of the vicinal hydrogen sites efficiently removes the effect of ^3^*J*_HH_ coupling, resulting in ‘normal’-looking relaxation dispersion profiles (Fig. 3; blue symbols). Y33ε, F52ε, and W43ζ3 (Figs. 3b, d, f) show flat dispersion profiles in the deuterated sample, again in agreement with expectations (very fast exchange, no ^1^H chemical shift difference between the symmetric sites, and no exchange, respectively; see above). In the case of F30ε, the site-selectively deuterated sample reveals a fast exchange process that is completely hidden by the strong-coupling effect in the protonated sample (Fig. 3c). Finally, for Y3ε where the relaxation dispersion profile shows clear signs of exchange even in the non-deuterated sample, the profile is more pronounced in case of site-selective deuteration (Fig. 3a). This comparison clearly shows that the Y3ε profile in the non-deuterated sample is compromised and cannot be analyzed to yield reliable exchange parameters. Additional, unspecific 70% background deuteration of the protein does slightly shift the ^1^H *R*_1*ρ*_ to lower values, but does not alter the profile shape (Fig. 3c, d, green symbols). A quantitative analysis comparing the results obtained from the samples with or without background deuteration shows that identical exchange parameters are obtained, within the margin of error (see below). This result demonstrates that site-selective deuteration in the aromatic ring alone is sufficient to obtain artifact free relaxation dispersion profiles, and further indicates that long-range ROE effects (Lundström and Akke 2005; Weininger et al. 2013a) do not cause concern in the present case.

Taken together, the present results illustrate the detrimental effects of ^3^*J*_HH_ couplings on the relaxation dispersion data obtained on non-deuterated samples, and clearly demonstrate that high-quality ^1^H *R*_1*ρ*_ relaxation dispersion profiles can only be obtained using samples with site-selective deuteration. Our present approach enables ^1^H *R*_1*ρ*_ relaxation dispersion experiments for Phe, Tyr, and Trp ζ3, and thus completes the repertoire of probes to cover all aromatic ^1^H-^13^C positions. By contrast, only Trp δ1, His δ2 and His ε1 are accessible also in non-deuterated samples, because these positions do not involve sizeable scalar coupling to vicinal protons.

### Hartmann–Hahn transfer explains artifacts in aromatic ^1^H R_1ρ_ relaxation dispersion experiments

**Fig. 4:**
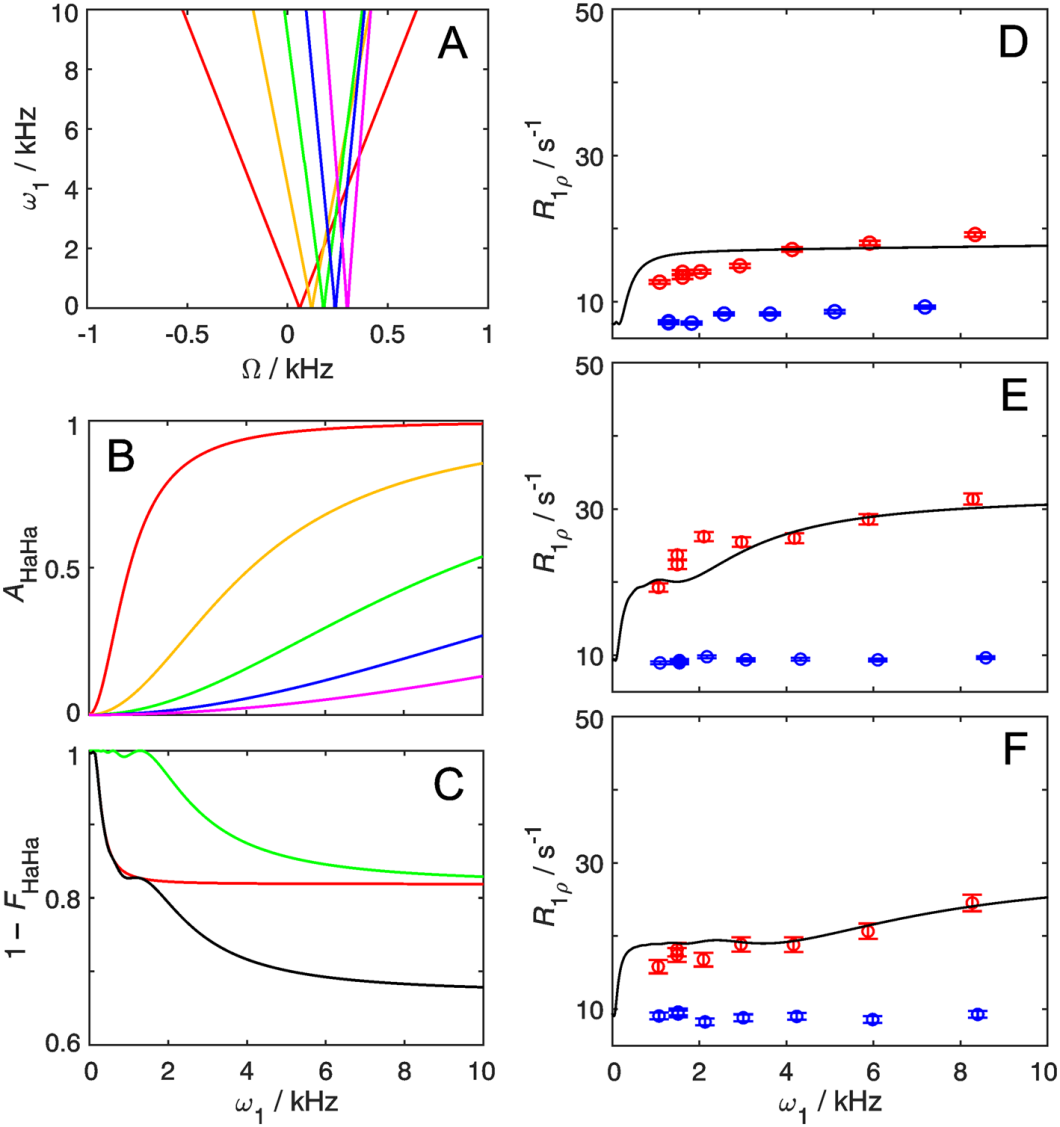
Hartmann–Hahn coherence transfer between spin-locked proton spins. The carrier frequency of the spin-lock is chosen to match the resonance frequency of spin *I*. **A** Contour levels of the coherence transfer amplitude, *A*_HaHa_ = 0.5, as a function of the spin-lock *ω*_1_ and the resonance offset from the spin-lock carrier for spin *S*, *Ω*. Contour lines are shown for various differences in resonance frequency offsets between the coupled nuclei: 0.2 ppm (red), 0.4 ppm (yellow), 0.6 ppm (green), 0.8 ppm (blue) and 1.0 ppm (magenta). **B** *A*_HaHa_ plotted as a function of spin-lock field strength for different resonance frequency offsets as in (**A**). **C** Loss of coherence according to the transfer function, 1 – *F*_HaHa_, for resonance frequency offsets of 0.2 ppm (red) and 0.6 ppm (green), and the product of both (black). **D**–**F** Measured data points from Y33ε (**D**), F52ε (**E**), and W43ζ3 (**F**) as given in Fig. 3, together with predicted apparent *R*_1*ρ*_ dispersion profiles (black lines), calculated using Eq. 6 and the artifact-free data measured on deuterated samples (blue points)

The artifacts described above for the non-deuterated samples (Fig. 3) can be rationalized as increased homonuclear Hartmann–Hahn transfer from the proton of interest (*I*) to a scalar coupled vicinal proton (*S*) with increasing spin-lock field strength (*ω*_1_). Figure 4 shows the expected values of the amplitude (*A*_HaHa_) of the transfer function and the relative loss of magnetization for spin *I* (1–*F*_HaHa_), as a function of the spin-lock field strength and the difference in offset between spins *I* and *S*; see Eqs. (3–5). The magnetization loss expected for the case where spin *I* has two *J*-coupled vicinal protons, as for Phe ε, is included in Fig. 4c; see Eq. (6). We calculated the amount of magnetization lost from *I* during the spin-lock period for those residues that do not show exchange contributions to *R*_1*ρ*_ (Y33ε, F52ε, and W43ζ3), and estimated their resulting apparent *R*_1*ρ*_ rate constant as a function of *ω*_1_. To do so, we took the average *R*_1*ρ*_ rate constant measured on site-selectively ^1^H–^13^C/^2^H–^12^C labeled samples to represent the artifact-free first term of Eq. (6), and added the second term involving the calculated value of (1–*F*_HaHa_). As shown in Figs. 4d–f, in all three cases the experimental data points acquired on the uniformly protonated sample are reproduced well by the calculated function describing the apparent *R*_1*ρ*_ values. Thus, Hartmann–Hahn transfer quantitatively explains the observed artifacts in aromatic ^1^H *R*_1*ρ*_ relaxation dispersion experiments acquired on uniformly protonated samples.

### Comparison of ring flip rates of ^1^H and ^13^C based aromatic R_1ρ_ relaxation dispersion experiments

**Fig. 5:**
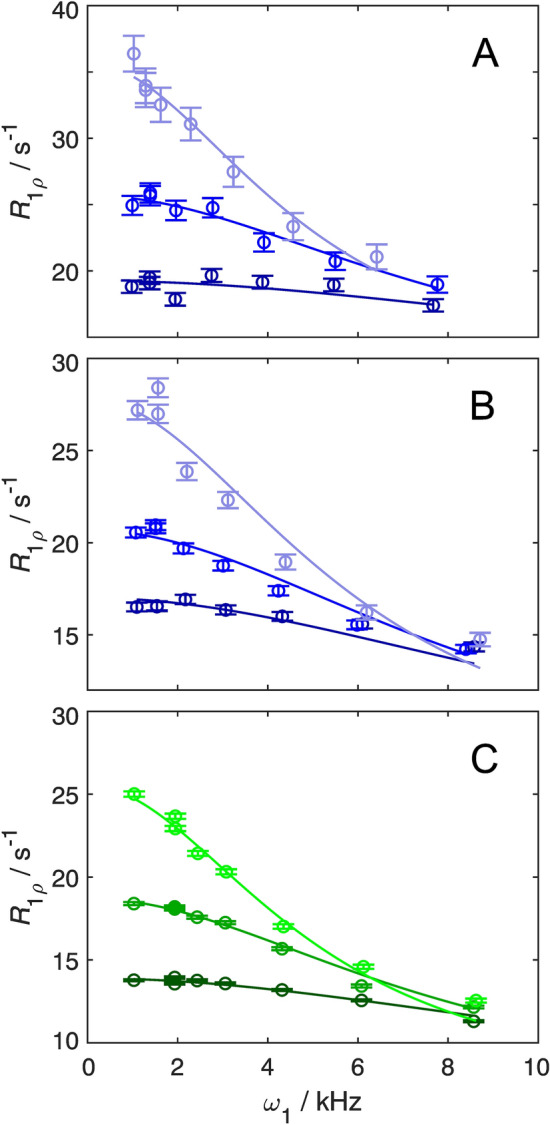
Temperature dependent aromatic^1^H *R*_1*ρ*_ relaxation dispersion data. Experiments were recorded on-resonance (*θ* = 90° from the z-axis) on site-selectively ^1^H–^13^C/^2^H–^12^C labeled GB1 at pH 7.0 and a static magnetic field strength of 14.1 T. Data of Y3ε acquired at 25 °C, 30 °C, and 35 °C and F30ε at 15 °C, 20 °C, and 25 °C are represented by light, medium, and dark hues, respectively. **A** Y3ε (blue), **B** F30ε (blue), and **C** F30ε with additionally 70% deuterated background (green). The relaxation dispersions were fitted using a fixed population *p*_1_ = *p*_2_ = 0.5 and *∆*δ as a free parameter with the restrictions: *k*_flip_ (*T*_high_) > *k*_flip_ (*T*_low_), *R*_2,0_ (*T*_high_) ≤ *R*_2,0_ (*T*_low_). The derived ring flip rate constants (*k*_flip_) are given in Table 1

**Fig. 6:**
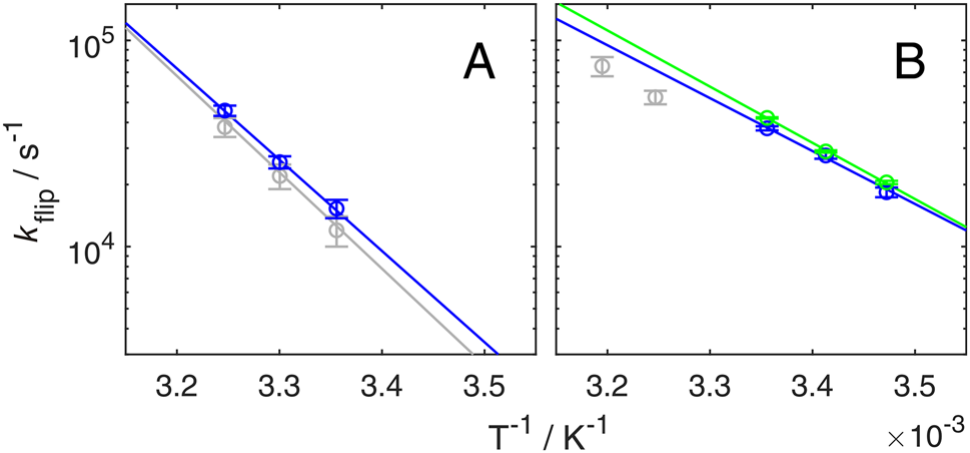
Temperature dependence of ring-flip rates. *k*_flip_ is plotted as a function of 1/*T*. **A** Y3ε and **B** F30ε flip rates determined from aromatic ^1^H *R*_1*ρ*_ relaxation dispersion measurements are shown in blue (protonated background) and green (deuterated background), and from previous ^13^C *R*_1*ρ*_ relaxation dispersion measurements in grey (Dreydoppel et al. 2020). Solid lines represent non-linear regression of *k*_flip_ on *T* according to the Eyring equation*,* displayed on a logarithmic y-axis to show the expected linearity. Derived activation enthalpies (∆*H*^‡^) and entropies (∆*S*^‡^) are: Y3 ^1^H (protonated background), (82 ± 8) kJ mol^−1^ and (111 ± 26) J mol^−1^ K^−1^ (**A**, blue); Y3 ^13^C (87 ± 14) kJ mol^−1^ and (126 ± 46) J mol^−1^ K^−1^ (**A**, grey); F30 ^1^H (protonated background), (47 ± 4) kJ mol^−1^ and (0 ± 13) J mol^−1^ K^−1^ (**B**, blue); F30 ^1^H (deuterated background), (50 ± 1) kJ mol^−1^ and (10 ± 4) J mol^−1^ K^−1^ (**B**, green)

We acquired relaxation dispersion profile for Y3ε and F30ε in the site-selectively deuterated sample at three different temperatures (Fig. 5). The artifact-free relaxation dispersions could be fitted to the general equation for symmetric exchange, resulting in flip rates at each temperature together with a global chemical shift difference for each residue (Table 1). The derived chemical shift differences are ∆*δ* = (0.46 ± 0.03) ppm for Y3ε and (0.47 ± 0.02) ppm for F30ε, in good agreement with the values measured in the spectrum under slow exchange conditions (0.50 ppm and 0.56 ppm, respectively). In the case of Y3ε, the resulting flip rates are virtually the same, within the margin of error, as those determined from ^13^C based experiments (Fig. 6a, Table 1). Consequently, the activation enthalpy (∆*H*^‡^) and activation entropy (∆*S*^‡^) determined from the temperature dependence of the flip rates are identical within the margin of error to previous estimates (Fig. 6a): ∆*H*^‡^ = (82 ± 8) kJ mol^−1^ for ^1^H versus (87 ± 14) kJ mol^−1^ for ^13^C; and ∆*S*^‡^ = (111 ± 26) J mol^−1^ K^−1^ versus (126 ± 46) J mol^−1^ K^−1^. In case of F30ε, we cannot directly compare flip rates determined by ^1^H and ^13^C *R*_1*ρ*_ experiments, because its ^13^Cε chemical shift difference is zero. However, ring flip rates have been measured for F30δ using ^13^C *R*_1*ρ*_ relaxation experiments at two higher temperatures, although the flip rate is far outside the optimal range of the *R*_1*ρ*_ experiment in this case and should only be interpreted semi-quantitatively (Fig. 6b). At lower temperatures F30δ becomes broadened beyond detection, because of its large ^13^Cδ chemical shift difference. The rates derived from the ^1^H *R*_1ρ_ experiments seem to be a bit higher (as gauged from the extrapolated line in Fig. 6b), although the two data points from the ^13^C *R*_1ρ_ experiments seem to follow the temperature dependence determined from the ^1^H *R*_1*ρ *_ experiments. Background deuteration in the F30ε sample leads to a minor improvement of the fit in the region of the highest refocusing frequencies (Fig. 5 bc), where the ROE effect is the most severe. The derived values of ∆*H*^‡^ and ∆*S*^‡^ with and without background deuteration are also identical, within the margin of error (Fig. 6b): ∆*H*^‡^ = (47 ± 4) kJ mol^−1^ for ^1^H with protonated background versus (50 ± 1) kJ mol^−1^ with deuterated background; and ∆*S*^‡^ = (0 ± 13) J mol^−1^ K^−1^ versus (10 ± 4) J mol^−1^ K^−1^. In summary, aromatic ^1^H *R*_1*ρ*_ experiments allow the determination of correct exchange parameters, provided that the aromatic ring is site-selectively ^1^H/^2^H labeled; further deuteration of the background is not needed.

**Table 1:** Ring flip rates and chemical shift differences

		Y3^a^	Y3^b^	F30^a^	F30^c^	F30^b^
*∆δ*(ppm)		0.46 ± 0.03	0.50	0.47 ± 0.02	0.49 ± 0.02	0.56
*k*_flip_ (10^3^ s^−1^)	15 °C			18 ± 1	20 ± 1	
	20 °C			28 ± 1	29 ± 1	
	25 °C	15 ± 2	12 ± 2	37 ± 1	42 ± 1	
	30 °C	26 ± 2	22 ± 3			
	35 °C	46 ± 3	38 ± 4			53 ± 4
	40 °C					75 ± 8

^a^Derived from aromatic ^1^H *R*_1*ρ *_relaxation dispersion experiments on position ε of site-selective deuterated samples with protonated background

^b^Taken from (Dreydoppel et al. 2020); chemical shift difference of position ε are derived from spectra under slow exchange conditions; ring flip rates are derived from aromatic ^13^C *R*_1*ρ*_ relaxation dispersion experiments on Y3 ^13^Cδ and F30 ^13^Cδ In the case of F30 the flip rates are clearly outside the optimal range of the experiment and should only be interpreted semi-quantitatively

^c^Derived from aromatic ^1^H *R*_1*ρ*_ relaxation dispersion experiments on position ε of site-selective deuterated samples with deuterated background

### Concluding remarks

There are multiple positions available in aromatic side chains that are suitable for studying exchange dynamics. However, it is not uncommon that certain sites do not experience sufficiently large ∆*δ* for a given nucleus, or experience exchange rates outside of the optimal relaxation dispersion window for a given nuclide (e.g., ^1^H or ^13^C). Thus, it is highly advantageous to be able to measure exchange using different nuclides, so as to probe as many sites as possible in a given protein. For example, our studies of ring flip dynamics in GB1 highlight the advantage of using both ^1^H and ^13^C based experiments:Y3 can be probed using all four positions (^1^Hδ, ^13^Cδ, ^1^Hε and ^13^Cε ), while F30 and F52 can each be probed using only a single probe (^1^Hε and ^13^Cε , respectively). Our present approach extends the available toolbox to make all aromatic positions accessible by NMR relaxation dispersion experiments using either CPMG or *R*_1*ρ*_ refocusing elements. The higher effective field strength available in ^1^H *R*_1*ρ*_ experiments is clearly of advantage in this context, and allows to reliably study exchange processes up to an exchange rate of about 80,000 s^−1^ (equal to a flip rate of 40,000 s^−1^).

## Data Availability

All data generated or analysed during this study are included in this published article.
